# Cortico-cortical paired associative stimulation: a novel neurostimulation solution for modulating brain connectivity and networks

**DOI:** 10.3389/fnins.2023.1336134

**Published:** 2024-01-08

**Authors:** Jack Jiaqi Zhang

**Affiliations:** Department of Rehabilitation Sciences, The Hong Kong Polytechnic University, Kowloon, Hong Kong SAR, China

**Keywords:** paired associative stimulation (PAS), transcranial magnetic stimulation (TMS), connectivity, neural network, neuromodulation

## Introduction

Transcranial magnetic stimulation (TMS) noninvasively modulates brain excitability. Despite its advancement in research and clinical utility, current interventional TMS protocols can only target a single brain region. When applying two sequential TMS sessions to different regions, the overall after-effect is found to be uncontrollable. For instance, Do et al. (2018) have used sequentially applied theta burst stimulation (TBS, a patterned form of repetitive TMS) in two brain regions in healthy adults, including dorsolateral prefrontal cortex-primary motor cortex (M1), ventral premotor cortex (PMv)-M1, and M1-M1; however, the after-effects seem largely unpredictable due to response variability and complex metaplastic interactions between two brain regions. Therefore, there is indeed a lack of a reliable and robust neurostimulation protocol for modulating interregional connectivity and networks.

Paired associative stimulation (PAS) is a form of neuromodulation using TMS and peripheral nerve stimulation (Stefan et al., 2000). Its cellular mechanism is based on spike-timing dependent plasticity (STDP), i.e., if an input spike to a neuron tends to occur immediately before that neuron's output spike, then the connection is strengthened. If an input spike tends to occur immediately after an output spike, then the connection is weakened (Dan and Poo, 2004). PAS combines single pulses of electrical stimulation to a peripheral nerve (for stimulating the primary somatosensory cortex, S1) and single pulses of TMS over the contralateral M1. If the interval between the peripheral nerve stimulation and the TMS is 25ms (or individual N20^1^ latency), the S1 is activated immediately before the M1 activation, and the connection between S1 and M1 is strengthened (Stefan et al., 2000; Ziemann et al., 2004). When the interval is 10 ms (or individual N20 latency-5 ms), the activation of the S1 is always after the M1 activation, so their connection is weakened (Stefan et al., 2000; Ziemann et al., 2004). Research using PAS has demonstrated that a STDP-like effect can be induced in human M1-S1 connections.

**Figure 1:**
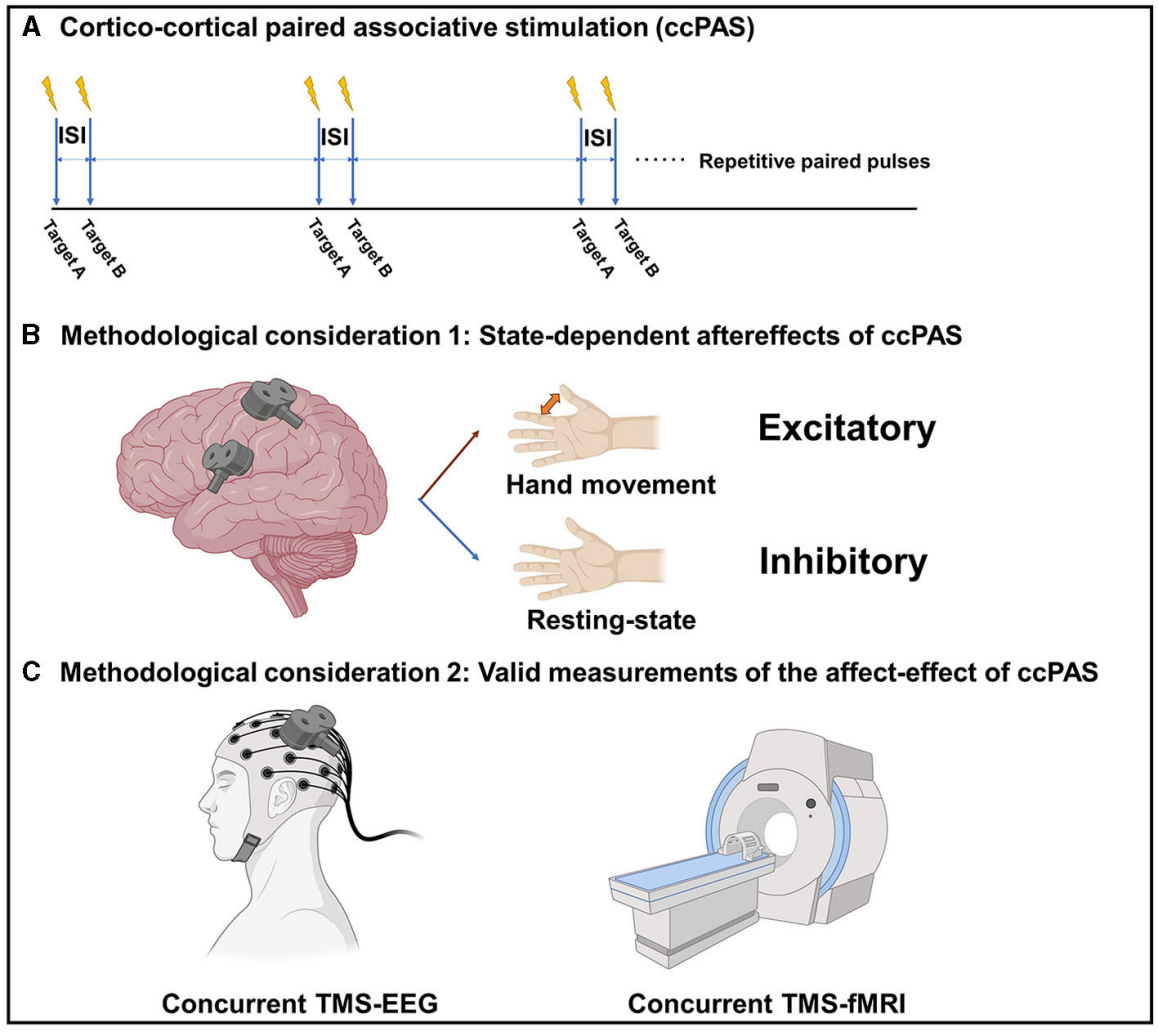
**(A)** The paradigm of cortico-cortical paired associative stimulation (ccPAS). **(B)** State-dependent after-effects of ccPAS. **(C)** Valid measurements of the after-effects of ccPAS. ISI, Inter-stimulus interval; TMS, Transcranial magnetic stimulation; EEG, electroencephalography; fMRI, functional magnetic resonance imaging. The figure was created with BioRender.com.

Cortico-cortical paired associative stimulation (ccPAS) differs from the ‘classical' PAS, which uses a dual-coil TMS approach to apply repetitive paired-pulse stimulations over two cortical regions (Guidali et al., 2021) (Figure 1A). ccPAS is believed to induce STDP-like plasticity over a cortico-cortical connection. Previous research has applied ccPAS paradigms in several cortico-cortical connections, such as M1-M1, PMv-M1, supplementary motor area (SMA)-M1, and posterior parietal cortex (PPC)-M1 (Guidali et al., 2021). The inter-stimulus interval is determined based on the estimated projection time between the two brain regions, in order to produce a bidirectional modulatory effect according to the rule of STDP.

## Discussion: methodological considerations of ccPAS

### State-dependence

The effect of ccPAS appears to be state-dependent. For example, it has been shown that the modulatory effect of PMv-M1 ccPAS is dependent on the current motor state. At resting state, ccPAS over the PMv and M1 could decrease the motor-evoked potential (MEP) in healthy adults, while ccPAS over the PMv and M1 tapped into motor tasks could increase the MEP in healthy adults (Davare et al., 2008; Buch et al., 2011). The reason behind this is that, during the resting state, the overall effect is inhibitory because the PMv-M1 glutamatergic projection activates the local inhibitory circuits more than the excitatory circuits within the M1. However, during movement status, the local inhibitory circuits are transiently blocked, so the projection activates the local excitatory circuits within the M1 therefore increasing the corticospinal excitability (Figure 1B). Therefore, the modulatory effect of ccPAS is likely to be bidirectional and depends on the current brain state.

### Valid measurements of the affect-effect of ccPAS

While it is assumed that ccPAS can modulate brain connectivity and networks, its modulatory effect has not been thoroughly examined using reliable techniques and biomarkers. Paired-pulse TMS-electromyography (EMG) measurements over a brain connection is the most commonly used outcome measure; however, they can only be applied to ccPAS that involves M1, and their output, in the form of a MEP, is not a pure cortical response. Indeed, we still need a valid measurement to assess the effect of ccPAS on modulating corticocortical connectivity and brain networks. Concurrent TMS-functional magnetic resonance imaging (fMRI) and TMS-electroencephalography (EEG) techniques are emerging cross-modal neuroscience measurement tools that may be used to study the modulatory effect of ccPAS on brain connectivity or networks. Upon perturbing a single brain region using TMS, the combined fMRI and/or EEG can capture the immediate and subsequent effects on the connected brain regions and the dynamics of neural networks, therefore making them unique solutions for evaluating brain connectivity and networks. There are also some pioneer works using concurrent recordings to study brain connectivity and networks. A concurrent TMS-fMRI study conducted by Bestmann et al. (2008) contributed a measurement of the connectivity between the dorsal premotor cortex (dPMC) and contralateral M1. Furthermore, the authors found that the facilitatory projection from the contralesional dorsal premotor cortex to the ipsilesional M1 was increased in patients after stroke with severe motor impairment in a compensatory manner (Bestmann et al., 2010). Additionally, a concurrent TMS-EEG study conducted by Bai et al. (2023) revealed a decrease in global efficacy when TMS stimulation was applied over the ipsilesional M1 compared to the contralesional M1 in stroke patients. This suggests an impairment of the brain network related to the ipsilesional M1 in chronic stroke. Thus, co-registration techniques enable the probing of brain connectivity and networks, potentially yielding a range of neural biomarkers for a comprehensive evaluation of the ccPAS effect (Figure 1C). It is of importance to note that a major limitation of concurrent TMS-fMRI and TMS-EEG is the contamination from non-neural sources in association with TMS. Therefore, a realistic sham condition is always required, which inevitably extends the measurement time (Gordon et al., 2018).

## Conclusion

ccPAS holds the promise to become a revolutionary neurostimulation paradigm for directionally modulating interregional brain connectivity and networks. It can serve a neuroscience research tool for studying the causal relationship between interregional connectivity and human behaviors. On the other, it could possibly become a novel therapeutic solution for repairing neural circuits, as various neurological and psychiatric conditions like stroke, Parkinson's disease, and major depressive disorders have been linked to impairments of brain connectivity and networks. Further study is encouraged to investigate the state-dependence of ccPAS to order to yield a robust modulatory effect and to establish a valid measurement for its affect-effect on brain connectivity and networks.

## Author contributions

JZ: Resources, Writing – original draft, Writing – review & editing.

## Footnotes

^1^N20 component of the somatosensory evoked potential.
